# Chemical Element Mixtures and Kidney Function in Mining and Non-Mining Settings in Northern Colombia

**DOI:** 10.3390/ijerph20032321

**Published:** 2023-01-28

**Authors:** Laura A. Rodriguez-Villamizar, Olga M. Medina, Oscar Flórez-Vargas, Eugenio Vilanova, Alvaro J. Idrovo, Santiago A. Araque-Rodriguez, José A. Henao, Luz H. Sánchez-Rodríguez

**Affiliations:** 1 Departamento de Salud Pública, Escuela de Medicina, Universidad Industrial de Santander, Bucaramanga 680002, Colombia; 2 Escuela de Microbiología, Universidad Industrial de Santander, Bucaramanga 68002, Colombia; 3 Instituto de Bioingeniería, Universidad Miguel Hernández de Elche, 03202 Elche, Spain; 4 Facultad de Ciencias de la Salud Programa de Medicina, Universidad Autónoma de Bucaramanga, Bucaramanga 681003, Colombia; 5 Escuela de Química, Universidad Industrial de Santander, Bucaramanga 680006, Colombia

**Keywords:** elements, trace elements, complex mixtures, kidney function, eGFR, mining, Colombia

## Abstract

The exposure to chemical mixtures is a problem of concern in developing countries and it is well known that the kidney is the major target organ for toxic elements. This cross-sectional study aimed to estimate the individual and composite mixture effect of a large number of chemical elements on kidney function in gold-mining and surrounding non-mining populations in northeast Colombia. We measured concentrations of 36 chemical elements in hair as indicators of chronic exposure from 199 adult participants. We estimated the effect of exposure to mixtures of chemical elements on estimated glomerular filtration rate (eGFR) using weighted quantile sum regression (WQS). The WQS index of the mixture was associated with reduced eGFR (Coefficient −2.42; 95%CI: −4.69, −0.16) being Be, Cd, Pb, As, and Mn, the principal contributors of the toxic mixture. Mining activities and Hg concentration were not associated with decreased kidney function. Our results suggest that complex mixtures of chemical elements, mainly heavy metals, act as nephrotoxic in these populations and therefore the analysis of chemical element mixtures is a better approach to identify environmental and occupational chemical risks for kidney damage.

## 1. Introduction

Chronic Kidney Disease (CKD) is characterized by a permanent loss of nephrons that lead to a progressive decline in glomerular filtration rate (GFR) [1]. It is estimated that CKD affects between 10 and 15% of the world’s population and its prevalence is rising along with the prevalence of other conditions such as diabetes, hypertension, and obesity [2]. Renal function is essential to homeostasis by removing excess water and waste from the blood, keeping a balance of chemicals in the body. However, in doing so, both tubular and glomerular kidney cells are exposed to metabolic wastes that include, among others, a wide variety of toxic chemical elements [3].

The kidney is the major target organ for toxic elements, mainly metals and metalloids, as urine is their route of excretion. The most known nephrotoxic elements are Pb, Cd, Hg and As [4,5,6], whereas some essential trace elements such as Se, Mn, and Zn are associated with protection of kidney function [7,8]. Humans are exposed to these elements from a variety of environmental, occupational, and dietary exposures which implies natural and anthropogenic sources [4,9]. Lee & Duffield reviewed some decades ago the main sources of 17 environmentally important metals (As, Be, Cd, Cr, Cu, Fe, Hg, Mg, Mn, Ni, Pb, Sb, Se, Sn, Ti, V, and Zn) and found that in the United States most of the emission sources were related to smelter/metallurgical processing, coal and oil combustion, and incineration [10]. Currently, less common chemical elements (i.e., Li, Ag, Ti, Ba) are used in innovative technologies of electronic hardware and nanomaterials, as well as in pharmaceutics, cosmetics, and agriculture industries and their presence in waters, food, and the human body may introduce a potential concern of their unknown toxic health effects [6].

The exposure to chemical mixtures is an increased matter of concern in developing countries. The presence of mines, small-scale enterprises, smelters, agricultural areas, and toxic waste disposal, among others, make the surrounding populations exposed to mixtures of a variety of metals, pesticides, and organic compounds [11]. In these countries, artisanal gold mining, which refers to the small-scale traditional gold-extraction process carried out using hand tools and chemicals, is responsible for one-tenth of all anthropogenic Hg emissions [12], estimating that about 13 million people around the world (including men, women and children) use the Hg-amalgamation process for gold recovery [13]. Since most studies in gold-mining populations have focused on adverse effects of Hg on health [14], where mainly Hg toxic effects on the kidney function were reported [15,16,17,18], the health effects of other metals and their chemical mixtures on kidney function remain much less known.

In Colombia, artisanal gold-mining activities using the Hg-amalgamation process has been practiced since the colonial period. There are 17 mining districts across the country, where the northeast and the northwest districts are the most productive [19]. The gold-mining district at northeast of the country include municipalities in which large-scale and artisanal gold mining are combined within the same territory. Our previous work on this mining population provided no support for early kidney damage associated with Hg vapor exposure in artisanal gold miners [20]. We also provided evidence that germline genetic variation, particularly in multispecific transporters and glutathione-related genes, mitigates Hg nephrotoxicity in miners [21,22]. However, the presence of reduced GFR in some people from the mining and mostly from the non-mining population is not fully explained by these genetic findings. In this context, we propose that there are other elements different than Hg that might be present in the environment of mining and non-mining surrounded areas that can enter the body through different exposure routes. Once in the body, those elements can produce a chemical mixture effect that might be biologically more important than the independent effect of each element. Thus, we hypothesized that mixtures of chemical elements (particularly heavy metals and metalloids) are associated with lower GFR.

In this study, we aimed to estimate the individual and composite mixture effect of a large number of chemical elements on kidney function in gold-mining and surrounding non-mining populations in northeast Colombia. We also aimed to assess if the effects of mixture on kidney function are associated with occupational gold-mining activities. We propose that the analysis of chemical element mixtures is a better approach to identify environmental and occupational chemical risks for kidney damage.

## 2. Materials and Methods

### 2.1. Study Design and Population

We conducted an observational analytic cross-sectional study in three municipalities of the department of Santander, located in the northeast mountain range of the Colombian Andes. Two municipalities belong to the northeast mining district of the country where the main economic activity is the exploration and extraction of gold (mining exposed group). The other municipality is located within the same geographical area of Santander (Soto Norte province) where the main economic activity is agriculture and other local activities not related to mining. According to the National Population Census 2018, the total population was 1762 and 1784 for the mining populations, and 2814 for the non-mining population for a total 6360 inhabitants in the three municipalities, with most of their population (63–75%) living in rural areas [23]. The mining towns are located around 7.33° latitude north and 72.94° longitude west, within an altitude range between 2000 m and 3350 m above sea level (asl), being one of them the municipality with highest altitude in Colombia. The non-mining town is located at 7.28° latitude north and 72.96° longitude west, with altitude of 2185 m asl. All three municipalities are located within an area that corresponds to a geological zone with high metallic content combined with high and low sulphuration deposits [24,25,26]. The study area is located within a range of 40–70 km northeast of Bucaramanga, the capital city of Santander.

### 2.2. Participant Recruitment

Participants from a previous cross-sectional study conducted in 2013 within the same municipalities were invited to participate if they met the selection criteria. The objective of that study was to assess the effects of chronic exposure to Hg in artisanal gold mining settings on biomarkers of renal dysfunction comparing non-exposed and exposed groups of individuals. Selection criteria and population characteristics were previously described in detail [20]. In that study we assessed the effects of Hg on health as this metal has been widely used by artisanal miners to extract gold using amalgamation procedures [27].

For the current study, the selection criteria for participants were as follows: for the gold-mining municipalities, the participants were adults, permanent residents in the municipality, and those who, during the last year, had any contact with Hg or mining-related activities. For the non-mining municipality, the participants were adult permanent residents with no history of contact with Hg or gold-mining activities. We excluded pregnant women and people with neurological disabilities who were not able to provide an interview or informed consent. Any exposure to chemical elements related to mining were captured by these inclusion criteria in the gold-mining municipalities while any other exposure to chemicals related to environmental exposure were captured by enrolling participants in mining and non-mining municipalities.

Participants were selected with a non-probabilistic sampling procedure based on selection criteria and willingness to participate. A random selection procedure was not feasible due to the lack of a sampling framework and the logistic limitation related to difficult geographical access to the municipalities. In the mining municipalities, participants who met inclusion criteria were included regardless of experience working in mining companies. In both mining and non-mining municipalities, invitation to participate was made by written flyers and voice to voice messages supported by community leaders and mining workers leaders. Recruitment of participants was conducted during the second semester of 2019.

### 2.3. Element Mixture Exposure: Sampling and Element Measurement

Hair samples from participants were used to account for biomarkers of chronic exposure to chemical elements [28,29]. The samples were collected from the occipital region of the scalp (250 mg) and stored in polypropylene bags with a previously assigned code for each participant. Each sample was placed in a plastic flask with a 100 µm nylon sieve on the bottom. Then, they were immersed in a distilled water solution containing 2% Triton X-100 in an ultrasonic bath during 1 min. Hairs were then washed by immersion in distilled water baths for 3 cycles of 1 min of duration and left for drying in an oven with dry air at 30 °C overnight. An amount of about 200 mg of hair was weighted in a 15 mL polypropylene conic tube and 1 mL of ultrapure concentrated nitric acid (65%) was added to the samples. Digestion was left at room temperature (25–28 °C) during more than 48 h with the cap not completely closed to permit air flux. The samples were diluted to a final volume of 10 mL. Then, a 10% volume of a solution with 100 μg/L Sc and Y was added to obtain a final concentration of 90.9 μg/L for Sc and Y as internal standard.

We measured 36 elements (essential and non-essential) that include Al, Ag, As, Au, B, Ba, Be, Bi, Ca, Cd, Ce, Co, Cr, Cu, Fe, Ga, Hg, K, Li, Mg, Mn, Mo, Na, Ni, Pb, Rb, Re, Sb, Se, Sn, Sr, Ti, Tl, V, W, and Zn. The elements were detected by Inductively Coupled Plasma Mass Spectrometry (ICP-MS). We used an Agilent 7500-A model with the software ChemStations G1834B, version B.03.02 (U300-0009) running on Microsoft^®^ Windows 2000^®^. The precision was monitored by including reagent blank, samples in duplicate, and certified standards. Certified multi-element and single element standard stock solutions were obtained by commercial suppliers as described in Supplementary Table S1. The accuracy of the method was verified on certified reference human hair materials (CRMs) for a total of 22 out of the 36 elements: Ag, As, B, Ba, Be, Bi, Ca, Cd, Co, Cr, Cu, Fe, Hg, Mg, Mn, Na, Pb, Sb, Se, Tl, V, and Zn (Supplementary Table S2)

To construct the calibration curve, solutions with different concentrations were prepared by serial dilution 1:3 with 5% nitric acid. For all preparations (i.e., blanks, standards, CRMs and samples), a volume of the solution of internal standard (Sc, Y 100 μg/L) was added in the proportion of 1/10/*v*/*v* before using the ICP-MS. For elements verified with CRMs, when measured concentrations were below the limit of quantification (LOQ), a value of half LOQ was assigned. For other elements, when measured concentrations were not detected we imputed to be 0 for statistical analysis. For the elements Au, Ce, Re, Sn, and Ti with no certified standards, the analysis was conducted with a preparation of a mixture of known concentration of other elements. While this strategy does not provide the actual absolute value of the estimated concentration, it can be used for comparing the relative values of concentration among the sub-populations of the analyzed samples. All procedures related to sample preparation and ICP-MS measurement were conducted at the Institute of Bioengineering at the Miguel Hernandez University of Elche (Elche, Spain).

### 2.4. Assessment of Kidney Function

Blood samples from participants were drawn into vacutainer tubes containing clot activator and separator gel but no anticoagulant to obtain serum by centrifugation at 3500 r/min for 10 min. We quantified creatinine in serum by the kinetic Jaffe method by using the Randox CREA kit along with the quality control reagent level 2 (Randox Laboratories Ltd., Crumlin, UK) on the Selectra JR clinical chemistry analyzer (Vital Scientific, Spankeren, Netherlands). Then, we calculated the estimated glomerular filtration rate (eGFR) using the equation from the Chronic Kidney Disease Epidemiology Collaboration (CKD-EPI) without race-adjustment as recommended for epidemiological studies [30,31] as follows: eGFR (mL/min/1.73 m^2^) = K1 × (serum creatinine/K2)^−1.209^ × 0.993^age^; where K1 = 144 and K2 = 0.7 if female or K1 = 141 and K2 = 0.9 if male [32].

### 2.5. Covariates

We used a structured questionnaire at the time of obtaining hair and blood samples for all participants to collect sociodemographic data, smoking status, and type of occupational activities, including mining activities. We categorized smoking status as current smoker (yes/no) and, when current smoking was stated, we recorded the number of cigarettes smoked per day. We calculated the body mass index (BMI) using the height and weight at the time of sample collection. After this procedure, participants were given an appointment with a medical general practitioner to complete a personal health history including a self-report of chronic diseases (i.e., diabetes, hypertension, and cancer) previously diagnosed by a physician and complete a general physical examination.

### 2.6. Statistical Analysis

We used descriptive statistics and dispersion measures to characterize the study population. Normality testing was conducted by using histograms and Shapiro–Wilk test. The eGFR showed a normal distribution. We log10-transformed hair element concentrations to obtain normal distributions and used transformed data for further analysis. We assessed correlation among elements using Spearman correlation coefficients.

We calculated quintiles of all log10-transformed data and used linear regression models to assess the association between quintiles of individual elements with eGFR as a measure of kidney function. Then, we used Weighted Quantile Sum Regression (WQS) [33] to assess the effect of the complex mixture of elements on eGFR. The WQS regression is a method commonly used in environmental epidemiology to assess the impact of chemical mixtures in relation to a health outcome of interest. WQS consists of two consecutive steps: first, a weighted index of standardized (using quantiles) concentrations is estimated using a nonlinear model where the final index is averaged across results of bootstrapping samples of observations or random subsets of components. Second, the regression coefficient of the weighted index is tested for statistical significance. The two steps are usually conducted in random splits of data with a specific portion of data used for weight estimation and the remaining used for hypothesis testing [34].

We used the WQS procedure with the following parameters: a Gaussian regression, with quintile exposure for all elements; 40% of data for weight estimation and 60% for hypothesis testing; a total of 1000 bootstraps for weight estimation; and an assumption of a positive coefficient to be false since we hypothesized that overall higher hair elements concentrations would be related to lower eGFR. We used the repeated holdout validation with random subset option of the WQS [35] or continuous data to obtain a better discrimination of the most important elements of the mixture and better estimations of effect under a situation of limited number of observations. By using this approach, the data are randomly partitioned 100 times, producing a distribution of validated results, and the mean is taken as the final estimate and its confidence intervals are estimated for inference [35]. We estimated the coefficient of the weighted mixture index adjusting for age, sex, mining activity, smoking status, BMI, and personal clinical history of diabetes and hypertension, as they are the main potential confounding factors of the association between chemical mixture and kidney function. We selected these variables for controlling for confounding oriented by a directed acyclic diagram (See Figure S1). Plots of the contribution of the elements to the chemical mixture were generated and a sensitivity analysis using different seeds was conducted for the WQS processing in order to enhance the robustness of the results [36]. We used the WQS R package version 3.0.4 for analysis [37].

## 3. Results

### 3.1. Characteristics of the Study Participants

A total of 199 participants were included in the present study (112 from mining and 87 from non-mining municipalities). The characteristics of the participants are shown in Table 1. All participants provided hair and blood samples and answered the general questionnaire. The medical clinical interview with chronic diseases assessment (i.e., previous diagnosis of diabetes and hypertension) was completed by 133 participants (66.8%; 75 from mining and 58 from non-mining municipalities). The lockdown and pandemic isolation measures occurred soon after the application of the first general questionnaire and before the scheduled medical interview. Once the pandemic isolation measures were lifted, we scheduled new appointments for medical interview. The 66 participants who missed the appointment with a medical general practitioner were not available after three consecutive attempts for setting up the medical clinical assessment. There were no statistically significant differences in basic characteristics and eGFR between participants who did and did not complete the medical clinical interview (See Table S3 in the Supplementary Materials).

The prevalence of comorbidities such as diabetes and hypertension in the 133 participants who complete medical interview was 8.27% and 15.79%, respectively. The prevalence was higher in the non-mining municipality with no statistically significant difference among groups: medical history of diabetes mellitus was reported by 7 participants (11.67%) in the non-mining municipality and 4 participants (5.19%) in mining municipalities (*p* value = 0.167); medical history of high arterial blood pressure was reported by 12 participants (20.00%) in the non-mining municipality and 9 participants (11.69%) in mining municipalities (*p* value = 0.167). Overall, four participants (2%) had clinical chronic kidney disease with eGFR below 60 mL/min/1.73 m^2^; three of them belonged to the non-mining population. The prevalence of chronic diseases was within expected range for the general population.

### 3.2. Individual Element Exposure and Association with eGFR

**Figure 1:**
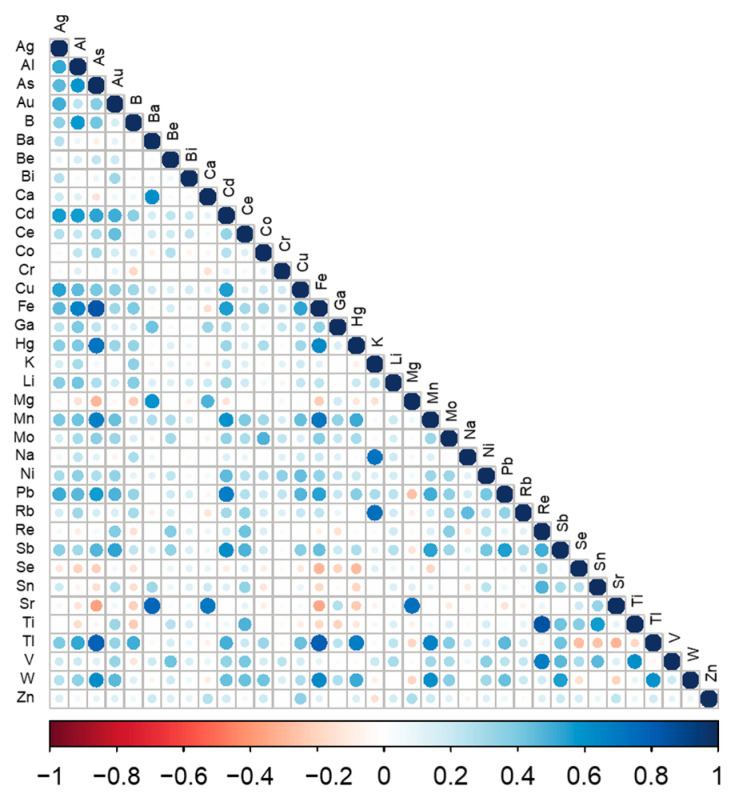
Correlation matrix of the 36 chemical elements concentrations measured in the hair of study participants. Color and size variation of circles in the figure are proportional level and direction (positive or negative) of the correlation between elements.

The distribution of detection and concentrations for all measured chemical elements are shown in Table 2. Ba and Hg were detected in all participants while Bi, Co, and Mo were detected in less than 8%. The highest correlations among elements were found between Re and Ti (Spearman ρ = 0.84), Fe and Tl (ρ = 0.80), As and Tl (ρ = 0.79), K and Rb (ρ = 0.73), Mg and Sr (ρ = 0.73); As and Hg (ρ = 0.72), K and Na (ρ = 0.72), Ca and Sr (ρ = 0.71), Fe and Mn (ρ = 0.71), Re and V (ρ = 0.69), As and Mn (ρ = 0.68), Cd and Pb (ρ = 0.68), Hg and Tl (ρ = 0.68), Al and Fe (ρ = 0.66), and Mn and Tl (= 0.66). Se was inversely correlated with Fe (ρ = −0.30), Hg (ρ = −0.28), As (ρ = −0.22), Al (ρ = −0.21), and Mn (ρ = −0.19) (Figure 1).

One quintile increase in element concentration was associated with a statistically significant decrease in eGFR only for Be (Coefficient: −0.038 95% CI −0.058; −0.017), Cd (Coefficient: −0.024 95% CI −0.045; −0.004), and V (Coefficient: −0.020 95% CI −0.039; −0.000) after controlling for age, sex, and mining activity (*n* = 199). In the same adjusted models, one quintile increase in element concentration was associated with a statistically significant increase in eGFR only for Cr (Coefficient: 0.020 95% CI: 0.000; 0.039). One quintile increase in element concentration was associated with a statistically significant decrease in eGFR only for Be (Coefficient: −0.049 95% CI −0.076; −0.023), after controlling for age, sex, mining activity, diabetes, hypertension, current smoking status, and BMI (*n* = 133). In the same adjusted models, one quintile increase in element concentration was associated with a statistically significant increase in eGFR only for B (Coefficient: 0.029 95% CI: 0.006; 0.53).

### 3.3. Association of Element Mixtures with eGFR

The mixture of elements in hair was associated with eGFR. As shown in Table 3, the models 1 and 2 using the 22 elements with CRM exhibited statistically significant association between the WQS index and eGFR. In the fully adjusted model (Model 2), one quintile increase in the WQS mixture index was associated with a decrease of 2.42 mL/min/1.73 m^2^ in the eGFR (95% CI: −4.69; −0.16). Models 3 and 4 using all measured elements also showed a negative coefficient but did not reach statistical significance.

**Figure 2:**
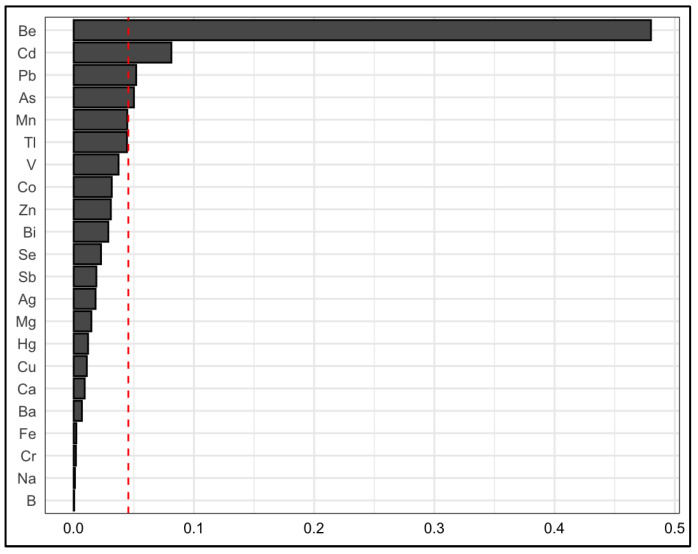
Weights of elements in the index mixture obtained by the weighted quantile sum regression (WQS) for the 22 elements with certified reference human hair materials and adjusted by potential confounders (Model 2). The red line shows the cut-off value of 0.045 for defining the elements with significant weights in the mixture index.

The chemical elements with significant weights in the toxic mixture that were present consistently in all models were Be, Cd, Pb, and As. Be was the strongest contributor of the WQS index in all models. In model 2, the mean weights of single elements to the WQS index were for Be (0.48), Cd (0.08), P (0.05), As (0.05), and Mn (0.045) (Figure 2).

The mining activity was not statistically associated with eGFR in any of the models. Age was inversely associated with eGFR and male sex and positively associated with eGFR in all models. The other covariates did not exhibit statistically significant associations with eGFR (Table 3).

## 4. Discussion

In this study, we found that a toxic mixture of chemical elements was associated with reduced eGFR in participants from gold-mining and surrounding non-mining populations. The principal contributors of the toxic mixture were Be, Cd, Pb, and As, while Hg concentration and mining activities were not associated with decreased kidney function. To our knowledge, this is the first analytical study conducted in adult population from mining surrounding areas assessing the effect of chemical mixtures on kidney function. Our results suggest that complex mixtures of chemical elements, mainly heavy metals, act as nephrotoxic in these populations.

Our findings regarding the nephrotoxic effect of Cd, Pb, and As are consistent with the broader literature that provide evidence of the individual adverse effect of these heavy metals on kidney function in humans and animals [3,4,6,38,39]. For example, in the United States higher Pb exposure was associated with reduced eGFR, whereas Cd exposure was associated with changes in blood urea nitrogen in a cohort study in premenopausal healthy women [40]. Similarly, higher levels of Pb were associated with reduced eGFR in adults from a population-based cohort in Sweden [41]. In Mexican children, increased levels of As and Cr were associated with urinary kidney injury molecule 1 (KIM-1) [42].

Our findings related to the main contribution of Be in the nephrotoxic mixture are not frequent in scientific literature. The nephrotoxicity of Be has not been reported before in general or gold-mining populations. In the past, many studies have associated Be exposure with many acute and chronic health outcomes, but mostly pulmonary affections, where the lung damage and immunological response lead to chronic beryllium disease; a granulomatous pulmonary disease was particularly described in workers from aeronautics industries [43,44,45]. There are, however, some occupational studies with workers exposed to Be and renal disease. A report from a cohort study in Be processing plant workers in the United States that reported an association between Be exposure and increased risk of chronic and unspecified nephritis, renal failure, and renal sclerosis [46]. In our study population, the association between higher levels of Be with reduced eGFR was statistically significant in both individual regression models and in all WQS mixture models, suggesting an important role of Be in kidney filtration measurements. Experimental studies with animal models have explored nephrotoxicity development from oral, inhalation, and intraperitoneal exposure to Be [47]. Although, in monkeys, the glomerular degeneration is not consistent with what has been observed in other animal species [48]. However, these findings should be interpreted with caution as overall Be concentrations were very low and one third of the sample had levels below the LOQ. The environmental sources of Be and its effect on kidney and lung function merit further investigation in our study population.

The nephrotoxic effects of mixtures of metals have been reported recently in general population of adults and children. In Southern China, higher concentrations mixtures of As and Mo in plasma of adult patients attending a hospital were associated with mild renal impairment [49]. Another study in Chinese adults with diabetes showed that higher concentrations of metal mixtures in urine, particularly As and V, were associated with chronic kidney disease [50]. In the United States, two studies of metal mixtures and kidney function have been reported. In adults over 40 years old, it was found that Pb levels and their interaction with Co had the strongest association with reduced eGFR when assessing a mixture of four heavy metals (Co, Cr, Hg and Pb) [51], whereas in adolescents it was found that Hg and Cd concentrations were associated with higher eGFR when analyzing a mixture of Pb, Cd, Hg, and As [52]. In Mexico, levels of Pb, Cd, and As were measured in urine and blood from adolescents from the PROGRESS longitudinal birth cohort study. This analysis found that the metal mixture in urine and blood are associated with increased eGFR and decreased cystatin C levels and that Cd and Pb are the main contributors of the mixture effects [53]. In addition, an analysis that included Cr and Li in the tooth-matrix from children from the same cohort found that exposure to a mixture of elements during the second and third trimester of gestation was the main window of vulnerability associated with children’s decreased eGFR with Li and Cr as the main contributors of the mixture effect [54].

The exposure to chemical mixtures is an emerging problem in developing countries where combinations of mining, agriculture, and toxic waste-disposal activities are present; as a consequence, some populations are exposed to mixtures of a variety of chemical elements, with evident environmental injustice as generally the most deprived socioeconomic and rural populations are the most exposed [11]. In Sub-Saharan Africa, heavy metal mixture exposure is considered a public health concern since artisanal mining activities, illegal refining, leaded petrol, among other anthropogenic activities, combined with deficient environmental legislation and policies, have posed a risk in the population’s health in recent decades [55]. In agricultural communities in the Americas and South Asia, the CKD of unknown origin is an emerging global concern that include as potential drivers the mixtures between metals such as Cd, As, Pb, and V, and herbicides such as glyphosate [56].

Mining settings are places where chemical mixtures have a higher probability to occur as elements in natural matrix (i.e., soil or water) and might be combined with elements from anthropogenic exploration and extraction procedures. Recently, a study conducted in artisanal Hg mining workers from Mexico showed that early kidney damage biomarkers were correlated with mixtures of Hg, Pb, As, and polycyclic aromatic hydrocarbons (PAHs) [57]. This is the only study we know so far that addressed the exposure to complex mixtures and its relationship with kidney damage in mining settings. For the study, Hg, As, Toluene, and PAHs were measured in urine samples and Pb was measured in blood samples, indicating acute chemical exposures. The analysis was exploratory in nature and used Spearman correlation to determine that all kidney function biomarkers had significant correlations with at least one of the chemical elements evaluated. Difference in our findings regarding lack of the association between Hg and kidney function might be explained by the level of exposure to Hg (reported to be very high in the mining workers in Mexico), the type of kidney function biomarker evaluated, the presence of genetic polymorphisms protecting against kidney damage in our population [21,22], and the statistical methods used in our study for assessing the effect of mixtures.

Our study adds evidence to the literature showing the adverse effect of element mixtures on kidney function by conducting an analytical study using specific statistical methods for assessing the effect of mixtures of elements on health. In addition, our study included gold-mining and non-mining workers from the surrounding population living in places with similar geological characteristics and used hair samples to account for chronic exposure to chemical elements in the environment. Thus, our study provides evidence of the risk of exposure to metal mixtures on kidney function not only in mining workers but also in general population from mining-influence areas.

The potential sources of exposure to metals in the study population might include contaminated water or food and occupational activities. This mining district is rich in Au and Ag, and the presence of hydrothermal zones with high quantities of minerals in soil such as Cu, Mo, Pb, Zn, As, Sb, and Hg have been described before [25]. Additionally, there are reports of variable zones with high and low sulphuration and different dominances of Au–Ag–Pb [26]. The potential mechanisms of action by which heavy metals produce kidney damage have been reported in humans and animals [58,59]. The common unifying mechanism is oxidative damage induced by the alteration of the mitochondrial electron transport chain that leads to production and accumulation of reactive oxygen species [60]. Oxidative DNA damage and inhibition of DNA repair are the main mechanisms underlying As genotoxicity. The accumulation of metals inside the mitochondria also produces vacuolation, lipid peroxidation, and its binding to thiol-containing proteins, causing impairment of protein function [7,56,60,61]. There is evidence that supplementation with Se, a powerful antioxidant essential element, can reduce the toxicity induced by As and Cd in humans and animals [7].

Our study has many strengths that are important to mention. First, we measured many chemicals that include essential and non-essential elements by using the same ICP-MS method. Despite certified hair materials being available for 22 out of 36 elements, we were able to quantify concentration for all elements, know their distribution in the study population, and include them in the statistical analyses. Second, we used hair samples of participants as a biomarker of chronic exposure, which is the main interest in long-term exposure assessment. In contrast to urine and blood samples which represent acute exposures, the use of hair has some advantages as it is a metabolic product that reflects the body metal burden of long-term exposure. Hair analysis assesses the extent of exposure to some metals and metalloids which tend to accumulate in the hair matrix by bonding to sulfur present in the keratin. The usefulness of hair as a biomarker tool has been proved under both physiological and pathological conditions since there is a correlation between element concentrations in this matrix and those found in the body [28,29,62,63,64,65]. For Be, the hair has been widely used as biomarker in human general population [66], and there is also animal experimental evidence supporting that hair is a good bioindicator of Be after inhalation exposure [67]. Third, we used WQS which is a relatively novel method for epidemiological analysis of mixtures; this method has the advantage of allowing the estimation of the overall mixture effect and the identification of the main toxic agents of the mixture [68,69]. Taken together, we were able to measure multiple chemical elements in hair and use epidemiological novel approach to better understand the complex mixtures that occur in the context of our study population and its effects on kidney function.

An important limitation of our study is its cross-sectional design that does not allow assessing the temporality of the association between chemical mixture exposure and kidney function. There might be a potential reverse causation in cross-sectional studies in which kidney damage will produce increased concentration of body elements. However, the low prevalence of clinical chronic kidney disease in our population makes this a less likely explanation for our findings. Prospective studies of exposure to multiple chemical elements in occupational settings and the general population will be needed to clarify the role of mixtures in early kidney damage and the variation of its effect with fluctuation in exposure. We did not collect detailed food questionnaires to assess the contribution of diet to concentration of elements in hair. However, the dietary intake of metals is part of its environmental exposure and by controlling for diabetes, hypertension, and BMI their potential confounding effect might not be affecting our estimations (see causal diagram in Supplementary Materials). The limited sample size of the study might limit the power to identify positive associations and might produce unstable effect measures. We minimized this limitation by using WQS with repeated holdout validation and a random subset to obtain robust estimates; thereby, despite the relatively small sample size, our study found a statistically significant association with the mixture index. Finally, in this paper we analyzed the total element concentration in hair as a biomarker of exposure in a massive study of multiple elements but with no chemical speciation that might be linked to renal damage. The possibility of assessment considering speciation, either of oxidative stage or organic forms of elements would be an interesting approach that could be addressed in the future for specific elements identified as critical for occupational and environmental health.

## 5. Conclusions

Chemical element mixtures, particularly mixtures of heavy metals, were associated with reduced eGFR in mining and non-mining populations in northern Colombia, independently of mining activities, sociodemographic characteristics, and chronic diseases. These findings are like those observed in some other regions of the world, which show that evaluating the isolated effect of toxicants is not enough to understand the dynamics of complex mixtures. There are synergistic effects between the different chemicals to which populations are exposed, and therefore the analysis of chemical element mixtures is a better approach to identify environmental and occupational chemical risks for kidney damage.

## Figures and Tables

**Table 1:** Characteristics of the study population.

Variable	All Participants *n* = 199	Non-Mining Municipality *n* = 87	Mining Municipalities *n* = 112	*p* Value
Male sex (*n*-%)	119 (59.80)	29 (33.33)	90 (80.36)	<0.0001
Age in years (mean-SD)	42.54 (12.04)	44.18 (11.66)	41.27 (12.22)	0.0901
Mining activities (*n*-%)	89 (44.95)	0 (0.00)	89 (79.46)	<0.0001
Years in current occupation (mean-SD)	12.92 (12.73)	14.87 (11.85)	11.40 (13.22)	0.0577
Current smoker (*n*-%)	16 (8.04)	4 (4.60)	12 (10.71)	0.115
BMI ^1^ (mean-SD)	26.98 (3.86)	27.52 (4.21)	26.56 (3.53)	0.0848
eGFR ^1^ mL/min/1.73 m^2^ (mean-SD)	88.68 (13.56)	85.62 (12.82)	90.86 (13.77)	0.0069

^1^ BMI = body mass index; eGFR = estimated glomerular filtration rate.

**Table 2:** Distribution of the hair concentrations of the chemical elements in participants (*n* = 199).

Element (ppb)	% Detection *	Median	Mean	SD	20th Percentile	80th Percentile
Elements verified with CRM						
Ag	96.98	0.15	1.46	5.16	0.03	0.9
As	91.96	0.11	0.97	1.86	0.02	1.58
B	90.96	1.19	2.79	4.29	0.25	4.58
Ba	100	0.85	1.32	1.57	0.043	1.92
Be	66.33	0.005	0.014	0.03	<LOQ	0.02
Bi	7.04	<LOQ	0.005	0.03	<LOQ	<LOQ
Ca	96.48	779.19	1018.31	1031.4	423.36	1331.69
Cd	63.32	0.008	0.087	0.33	<LOQ	0.076
Co	6.03	<LOQ	0.049	0.29	<LOQ	0.007
Cr	83.42	0.12	0.27	0.58	0.02	0.38
Cu	98.49	14.2	17.44	13.86	10.49	21.95
Fe	98.99	11.01	30.36	49.75	5.12	37.64
Hg	100	0.33	0.8	1.98	0.13	0.83
Mg	98.49	33.01	63.04	88.54	16.39	89.08
Mn	85.43	0.84	4.59	10.23	0.079	4.76
Na	98.99	127.54	209.25	232.22	69.92	299.32
Pb	98.99	1.48	7.82	35.46	0.53	6.26
Sb	55.28	0.0009	0.03	0.14	<LOQ	0.16
Se	84.92	0.58	0.6	0.59	0.14	0.93
Tl	94.47	0.003	0.029	0.072	<LOQ	0.031
V	75.88	0.053	0.075	0.081	<LOQ	0.13
Zn	99.5	214.81	252.55	178.18	161.56	300.45
Elements without verified CRM						
Al	92.96	11.22	20.72	25.18	3.6	36.44
Au	56.28	0.0009	0.04	0.33	ND	0.012
Ce	23.62	ND	0.001	0.002	ND	ND
Ga	89.95	0.005	0.008	0.014	0.002	0.009
K	97.99	66.6	101.04	119.73	28.54	145.22
Li	81.41	0.007	0.012	0.022	ND	0.018
Mo	6.03	ND	0.01	0.07	ND	0.015
Ni	76.38	0.1	0.41	2.62	ND	0.33
Rb	46.23	ND	0.047	0.091	ND	0.08
Re	54.27	ND	0.0004	0.0021	ND	ND
Sn	79.9	0.022	0.061	0.18	ND	0.08
Sr	97.49	0.1	2.35	3.74	0.41	3.95
Ti	51.26	0.028	0.058	0.078	ND	0.1
W	41.71	ND	0.014	0.045	ND	0.01

* Percent of the 199 samples with measurement above LOQ; LOQ = limit of quantification; ND = No detection in sample; CRM = certified reference human hair materials.

**Table 3:** Weighted quantile sum regression (WQS) results for the association between concentrations of chemical mixtures in hair and kidney function measured by estimated glomerular filtration rate (eGFR).

Variable	Mixture of 22 Elements with CRM ^1^				Mixture of All 36 Elements ^1^			
	Model 1 (*n* = 199)		Model 2 (*n* = 133)		Model 3 (*n* = 199)		Model 4 (*n* = 133)	
	Coefficient	95% CI	Coefficient	95% CI	Coefficient	95% CI	Coefficient	95% CI
WQS index	−2.13	−3.8 to −0.45	−2.42	−4.69 to −0.16	−1.85	−3.92 to 0.214	−2.11	−5.04 to 0.81
Male sex	4.35	0.71 to 7.99	4.69	1.02 to 8.35	3.58	−0.09 to 7.25	4.6	0.933 to 8.26
Age	−0.69	−0.81 to −0.58	−0.8	−0.97 to −0.63	−0.7	−0.82 to −0.58	−0.79	−0.97 to −0.62
Mining activity	2.8	−0.83 to 6.42	1.35	−2.56 to 5.26	3.05	−0.65 to 6.75	1.01	−2.97 to 5.00
Diabetes	NA		3.18	−2.17 to 8.53	NA		3.34	−1.99 to 8.67
Hypertension	NA		4.23	−0.46 to 8.91	NA		4.23	−0.43 to 8.90
Current smoking status	NA		−4.86	−11.60 to 1.82	NA		−5.01	−11.8 to 1.80
Body mass index	NA		−0.26	−0.69 to 0.18	NA		−0.27	−0.70 to 0.17
Main elements in mixture ^2^	Be, Cd, V, Tl, Ag, As, Pb, Co, Bi		Be, Cd, Pb, As, Mn		Be, V, Cd, Au, Tl, As, Ag, Pb, Re, W, Co, Mo		Be, Re, Au, Cd, Ga, Tl, As, Pb, Mn	

^1^ Models 1 and 3 partially adjusted by sex, age, and mining activity. Models 2 and 4 adjusted by age, sex, mining activity, diabetes, hypertension, smoking status, and body mass index. CRM = certified reference human hair materials. NA= Not Adjusted. ^2^ Chemical elements with significant weights in the mixture index using as cut-off value the inverse of the number of elements in the mixture: 0.045 in Models 1 and 2, and 0.028 in Models 3 and 4.

## Data Availability

Data availability upon request for specific purposes.

## Supplementary Materials

The following supporting information can be downloaded at: https://www.mdpi.com/article/10.3390/ijerph20032321/s1,: Table S1. Certified standards for trace elements concentration in human hair; Table S2. Limit of quantification (LOQ) and experimental values of trace elements concentration in the certified reference material of human hair; Table S3. Characteristics of participants who complete and did not complete clinical medical interview; Figure S1. Direct Acyclic Diagram (DAG) of the causal association between element mixtures in hair and estimated glomerular filtration rate (eGFR)
