# Supplementary material for: Chemical Element Mixtures and Kidney Function in Mining and Non-Mining Settings in Northern Colombia
Source: Int J Environ Res Public Health. 2023 Jan 28;20(3):2321. doi: 10.3390/ijerph20032321 (PMC9914985; doi:10.3390/ijerph20032321)

Supplementary material

Chemical Element Mixtures and Kidney Function in Mining and Non-Mining Settings in Northern Colombia

Laura A. Rodriguez-Villamizar [1,*], Olga M. Medina [2], Oscar Flórez-Vargas [2], Eugenio Vilanova-Gisbert [3], Alvaro J. Idrovo [1], Santiago A. Araque-Rodriguez [4], José A. Henao Martínez [5] and Luz H. Sánchez-Rodríguez [2]

[1] Departamento de Salud Pública, Escuela de Medicina, Universidad Industrial de Santander, Bucaramanga 680002, Colombia
[2] Escuela de Microbiología, Universidad Industrial de Santander, Bucaramanga 68002, Colombia
[3] Instituto de Bioingeniería, Universidad Miguel Hernández de Elche, 03202 Elche, Spain
[4] Facultad de Ciencias de la Salud Programa de Medicina, Universidad Autónoma de Bucaramanga, Bucaramanga 681003, Colombia
[5] Escuela de Química, Universidad Industrial de Santander, Bucaramanga 680006, Colombia
* Correspondence: laurovi@uis.edu.co

Contents

Table S1. Certified standards for trace elements concentration in human hair. 3

Table S2. Limit of quantification (LOQ) and experimental values of trace elements concentration in the certified reference material of human hair... 4

Table S3. Characteristics of participants who complete and did not complete clinical medical interview 6

Figure S1. Direct Acyclic Diagram (DAG) of the causal association between element mixtures in hair and estimated glomerular filtration rate (eGFR).. 7

Table S1. Certified standards for trace elements concentration in human hair.

Denominations	Company. Reference. Bath	Elements (Concentrations)
Multielement standard Solution 5 for ICP. TraceCERT	Sigma Aldrich 54794-100mL (BCBT5551)	27 elements in nitric acid 5%<br><br>Li, Be, Na, Mg, Al, K, Ca, V, Cr, Mn, Fe, Co, Ni, Cu, Zn, Ga, Rb, Sr, Mo, Ag, Cd, Cs, Ba, Tl, Pb, Bi, Hg
ICP multielement standard Solution X Certipur	Merck 1.09493.0100 (HC856557293)	23 elements in diluted nitric acid<br><br>Be, B, Na, Mg, K, Ca, V, Cr, Mn, Fe, Co, Ni, Cu, Zn, As, Se, Sr, Mo, Cd, Ba, Tl, Pb, Bi
Environmental Calibration Standard	Agilent. Part #5183-4688. (Lot#: 1-190YJY2	22 elements in nitric acid 5%<br><br>Na, Mg, Al, K, Ca, V, Cr, Mn, Fe, Co, Ni, Cu, Zn, Ga, Se, Mo, Ag, Cd, Sb, Ba, Tl, Pb,
Mercury ICP/MS standard CertiPUR	Merck 1.70333.0100 (hc02450533)	Hg 1000 mg/L. Very high concentration, Prepared Stock diluted at 10 mg/L
Aluminium ICP standard CertiPUR	Merck 1.70301.0100 (OC554360)	Al (as Al(NO3)3) in nitric acid 3% (Uses for confirmation purpose)
Scandium ICP/MS standard CertiPUR	Merck 1.70369.0100 (HC631658)	Sc 1000 mg/L in HNO3 7% Sc as Sc2O3). (Used as internal standards)
Ytrium ICP/MS standard CertiPUR	Merck 1.70369.0100 (HC631658)	Y 1000 mg/L in HNO3 7% Sc). (Used as internal standards)
Standard of high concentration of Na-K-Mg-Ca	Prepared in the lab from analytic grade reagents	Used to confirm cases with concentrations higher than 100 mg/L.

Table S2. Limit of quantification (LOQ) and experimental values of trace elements concentration in the certified reference material of human hair.

Certificate material	*Element*	*LOQ (ppb)*	*Experimental value (ppb) M ± SD*	*95% Confidence Interval*
NCS DC 73347 a	Na	11	78,23 ± 5,82	61 – 117
	Ba	0,02	12,03 ± 0,33	10 – 12.8
	Hg	0,01	0,463 ± 0,006	0,44 – 0,9
	Tl	0,001	0,0068 ± 0,0003	0,0051 – 0,010
	Bi	0,001	0,019 ± 0,001	0,016 – 0,026
	V	0,01	0,32 ± 0,02	0,09 – 0,91
	Fe	0,5	28,9 ± 2,1	24 - 48
	Cr	0.01	0,12 ±0,01	0,13 – 0,69
	Co	0.01	0.026 ± 0.002	0.024 – 0.066
	B	0.030	2,39 ± 0.15	1,75 – 4,05
	Be	0,001	0,1045± 0,003	0,094 – 0,126
IAEA 086	Mn	0,02	9,98 ± 0,3	8,8 ± 10,4
	Mg	0,5	162,42 ± 10	150 - 200
	Ca	29	1127 ± 35	1010 - 1230
ERM DB001	Cu	0,04	28,9 ± 2,2	24 - 42
	As	0,01	0,038 ± 0,02	0,03 – 0,06

	Pb	0,01	1,98 ± 0,02	1,7 – 2,6
NIES 13	Zn	0,2	170 ± 16	146 - 197
	Sb	0,01	0,034 ± 0,003	0,024 – 0,060
	Se	0,01	2,08 ± 0,2	1,4 – 2,2
	Ag	0,01	0,12 ± 0,03	
	Cd	0,01	0,24 ± 0,05	0,16 – 0,30

Table S3. Characteristics of participants who complete and did not complete clinical medical interview

Characteristic	Complete clinical assessment n=133	Missing from clinical assessment n=66	p value
Male (n-%)	75 (56.39)	44 (66.67)	0.164
Age (mean-SD)	43.51 (11.21)	40.57 (13.43)	0.133
Mining activities (n-%)	59 (44.36)	30 (46.15)	0.812
Current smoker (n-%)	10 (7.52)	6 (9.09)	0.701
BMI (mean-SD)	26.94 (3.79)	27.05 (4.04)	0.549
eGFR mL/min/1.73m2 (mean-SD)	87.95 (13.09)	90.13 (14.46)	0.348

Figure S1. Direct Acyclic Diagram (DAG) of the causal association between element mixtures in hair and estimated glomerular filtration rate (eGFR)

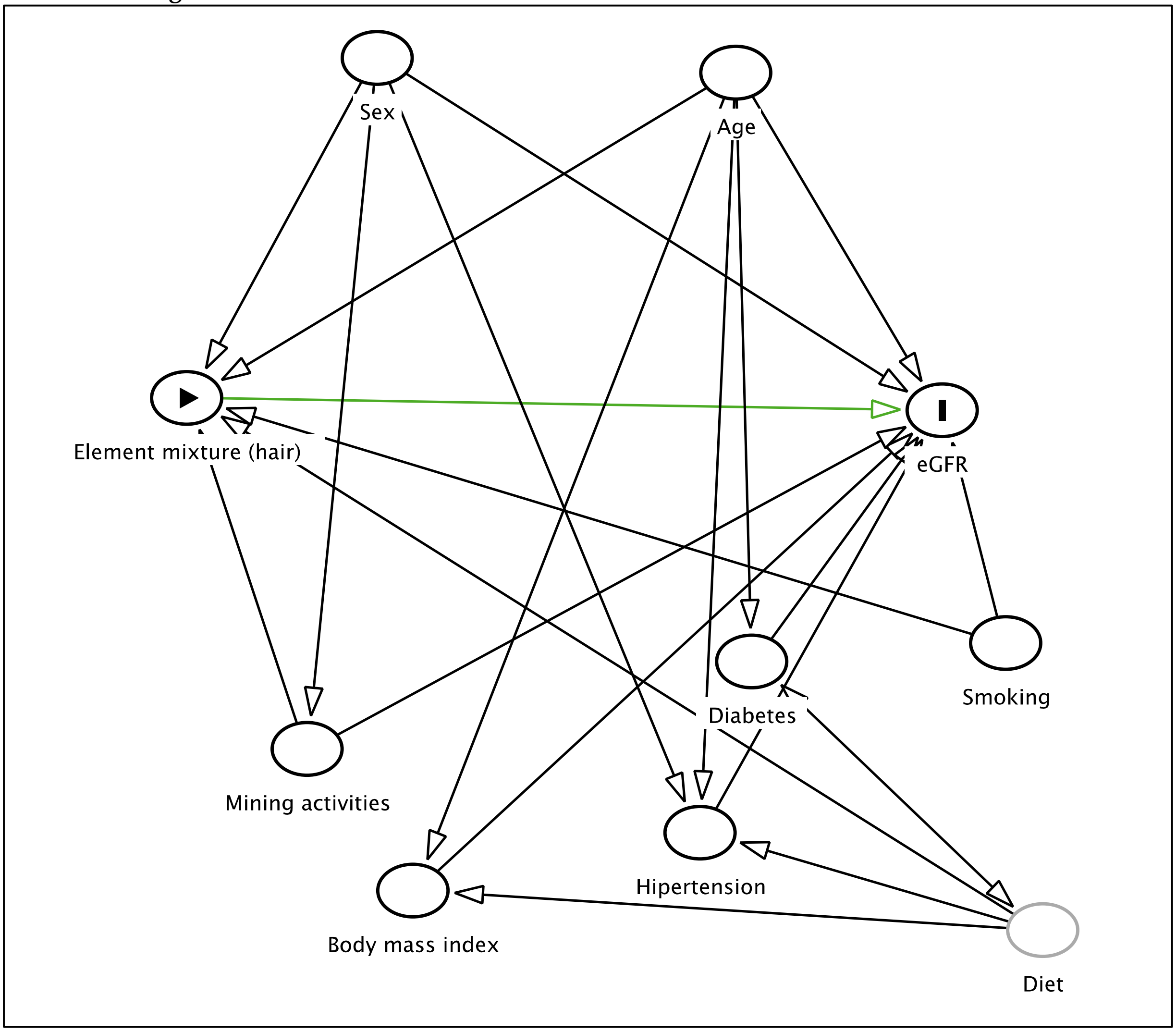

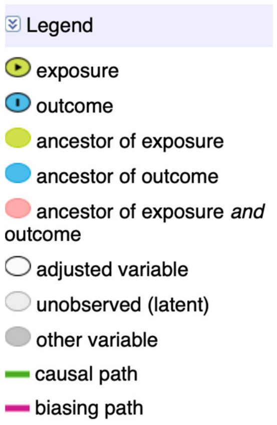

Supplement: Supplementary file 1 [file ijerph-20-02321-s001.zip › ijerph-2124984-supplementary.pdf]
